# Phenotypic and Genetic Profile, Biofilm-Forming Ability and Antibiotic Sensibility of ESBL-Producing *Klebsiella pneumoniae* Complex from Fecal Samples of Cats in Italy

**DOI:** 10.3390/antibiotics15080735

**Published:** 2026-07-29

**Authors:** Alessia Facchin, Gabriele Ratti, Irene Mauri, Alessia L. Gazzonis, Paola Dall’Ara, Claudia Pollera, Maria Cristina Rapi, Stefania Lauzi

**Affiliations:** 1 Department of Veterinary Medicine and Animal Sciences, University of Milan, Via Dell’Università 6, 26900 Lodi, Italy; alessia.facchin@guest.unimi.it (A.F.); gabriele.ratti@i-vet.it (G.R.); irene.mauri@unimi.it (I.M.); alessia.gazzonis@unimi.it (A.L.G.); paola.dallara@unimi.it (P.D.); claudia.pollera@unimi.it (C.P.); maria.rapi@unimi.it (M.C.R.); 2 I-Vet s.r.l., Via Ettore Majorana 10, 25020 Flero, Italy

**Keywords:** *Klebsiella pneumoniae* species complex, antimicrobial resistance, multidrug resistance, fecal shedding, pathotype, biofilm, One Health

## Abstract

Background: Antimicrobial resistance mediated by ESBL-producing *Klebsiella pneumoniae* is an emerging concern in both human and veterinary medicine, with companion animals increasingly considered relevant within the One Health framework. This study aimed to investigate the fecal carriage of ESBL-producing *K. pneumoniae* complex in cats from Italy and to characterize the strains by the phenotypic and genetic profile of ESBL production, virulent pathotypes, antibiotic resistance profile and biofilm production. Methods: Fecal samples collected from cats admitted to the Veterinary Teaching Hospital of Milan (Italy) in 2020–2026 were bacteriologically and genetically analyzed. Results: All the *Klebsiella pneumoniae* strains isolated [4/200 (2%, 95% CI: 0.06–3.94%)] were ESBL-producing *K. pneumoniae* complex isolates harboring *bla*_CTX-M-15_, *bla*_SHV_, and *bla*_TEM_ genes. The isolates were detected with higher presence in cats with diarrhea and were found only in cats treated with antibiotics and hospitalized. All four ESBL-producing isolates were classified as the classical *K. pneumoniae* pathotype based on the negative string test results, the absence of reliable virulence genes used for pathotype identification (*peg-344*, *iucA*, *rmpA* and *rmpA2*), and the lack of K1 and K2 serotypes, despite the detection of *terB* and *irp2* virulence genes in one and two isolates, respectively. All four ESBL-producing *K. pneumoniae* complexes were classified as multidrug-resistant, with resistance mainly observed to β-lactams, fluoroquinolones, quinolones and folate antagonists. All four ESBL-producing *K. pneumoniae* complexes demonstrated biofilm-forming abilities, with two isolates showing weak adhesion, one moderate adhesion, and one strong adhesion. Conclusions: The detection of ESBL genes together with the MDR pattern, biofilm-forming capacity and selected virulence determinants suggests the potential epidemiological relevance of cats in the dissemination of antimicrobial-resistant *K. pneumoniae* complexes, underscoring the need for strengthened surveillance and prevention strategies in veterinary settings to provide information to pet cat owners and children who may interact with stray cats, in full implementation of the One Health approach.

## 1. Introduction

Antimicrobial resistance (AMR) has been identified worldwide as one of the most urgent health threats [1], requiring the monitoring of specific antibiotic-resistant bacteria that belong to the global priority list published by the World Health Organization (WHO). The WHO has categorized third-generation cephalosporin (3GC)-resistant Enterobacterales as a critical priority group [2]. The resistance to 3GC is mediated principally by acquired extended-spectrum β-lactamase (ESBL) genes located on plasmids, which confer resistance to all β-lactams except for carbapenems and cephamycins. Moreover, ESBL-producing bacteria frequently harbor other genes that confer resistance to a wide variety of antimicrobial agents. ESBL production has been identified as the principal cause of drug resistance in Gram-negative bacilli, leading to great therapeutic challenges in humans and animals due to increased treatment failure, hospitalization, and mortality [2].

The WHO requires a One Health approach to combat AMR [1]. Although numerous studies have focused on ESBL/AmpC-producing *Escherichia coli* as commensal indicator microorganisms for AMR surveillance systems [1,3] in food producing animals and pets worldwide, raising concern that dogs and cats may be possible sources and reservoirs of ESBLs for humans [4,5,6], epidemiological gaps still need to be fulfilled in AMR surveillance and control. Indeed, in the past decade, the incidence of ESBL-producing *Klebsiella pneumoniae*, within the 3GC-resistant Enterobacteriaceae, has increased in humans [7]. *K. pneumoniae* along with *Enterococcus faecium*, *Staphylococcus aureus*, *Acinetobacter baumannii*, *Pseudomonas aeruginosa*, and *Enterobacter* species also belongs to the WHO list of priority pathogens with increased antibiotic resistance, known as ESKAPE [1]. Treatment in *K. pneumoniae* is complicated by both its resistance to multiple antibiotics, particularly through the production of ESBLs, and its ability to form biofilms [8].

*K. pneumoniae* is an important conditional pathogen in humans and animals. Approximately one-third of all Gram-negative infections in humans are attributed to *K. pneumoniae*, and this bacterium has been reported as a major human pathogen in both healthcare and community settings, responsible for various infections [9]. *K. pneumoniae* can be divided into classical and hypervirulent pathotypes based on virulence level and associated infectious disease. Hypervirulent *K. pneumoniae* (hvKP) causes severe community-acquired infections in healthy individuals and is characterized by the presence of virulent genes (with *peg-344*, *iucA*, *rmpA* and *rmpA2* genes showing high diagnostic accuracy for hvKP identification), whereas classical *K. pneumoniae* (cKP) is genetically heterogeneous in the absence of virulence-associated genes and is mostly associated with nosocomial infections in critically ill and immunocompromised patients [10].

As in humans, *Klebsiella* spp. in companion animals may occur in clinical settings, and the infection is often associated with clinical diseases, including diarrhea, respiratory and urinary tract infections [11,12,13]. Isolates from clinically ill dogs and cats have been detected in different countries from Asia, North and South America and Europe [13,14,15,16,17,18]. Moreover, besides the presence of ESBL-/AmpC-producing *K. pneumoniae*, isolates with resistance to carbapenem, considered last-resort antibiotics, have also been reported in pets [19]. Besides clinical samples, fecal carriage of ESBL-producing *K. pneumoniae* in healthy pets has been reported even if it has been rarely investigated [20,21]. The risk of transmission of ESBL/AmpC-carrying *K. pneumoniae* between companion animals and humans has also been supposed [11,21].

The emergence of ESBL/AmpC-producing *K. pneumoniae* is a significant concern in humans and veterinary medicine. Given that animals may contribute to its spread, the detection of ESBL-producing *K. pneumoniae* has become an important issue in veterinary medicine, especially in pets when also considering their fecal carriage, which may further contribute to dissemination. Despite being the most common companion animal worldwide, studies on *K. pneumoniae* infection in cats are more limited compared to dogs. To fill in some epidemiological gaps in AMR monitoring, the aims of this study were (i) to detect the presence of fecal carriage of ESBL-producing *K. pneumoniae* complex (which includes seven species, namely *K. pneumoniae* subsp. *pneumoniae*, *K. quasipneumoniae* subsp. *quasipneumoniae*, *K. quasipneumoniae* subsp. *similipneumoniae*, *K. quasivariicola*, *K. africana*, *K. variicola* subsp. *tropica*, and *K. variicola* subsp. *variicola*) in healthy and unhealthy cats, and (ii) to characterize the strains by the phenotypic and genetic profile of ESBL production, phenotypic characterization of virulent pathotypes and presence of virulence genes, antibiotic resistant profile and biofilm production in order to define potential risks of transmission.

## 2. Results

### 2.1. Animal and Sample Caseload

Cats were randomly selected from the total case load of cats admitted to the Veterinary Teaching Hospital (VTH) of Lodi, University of Milan, and a total of 200 fecal samples were collected. Analysis was performed on fecal samples. Cats’ mean age was 2 years and 9 months, with age ranging from 2 months to 14 years and 8 months. Out of the 66 unhealthy animals, 40 showed gastrointestinal (GI) symptoms, including 31 cats with diarrhea.

Data regarding the population characteristics of the cats analyzed are summarized in Table 1.

### 2.2. ESBL-Producing Klebsiella pneumoniae Complex Isolates

The distributions of positive results for the ESBL-producing *K. pneumoniae* complex according to the characteristics of cats are reported in Table 1.

**Figure 1:**
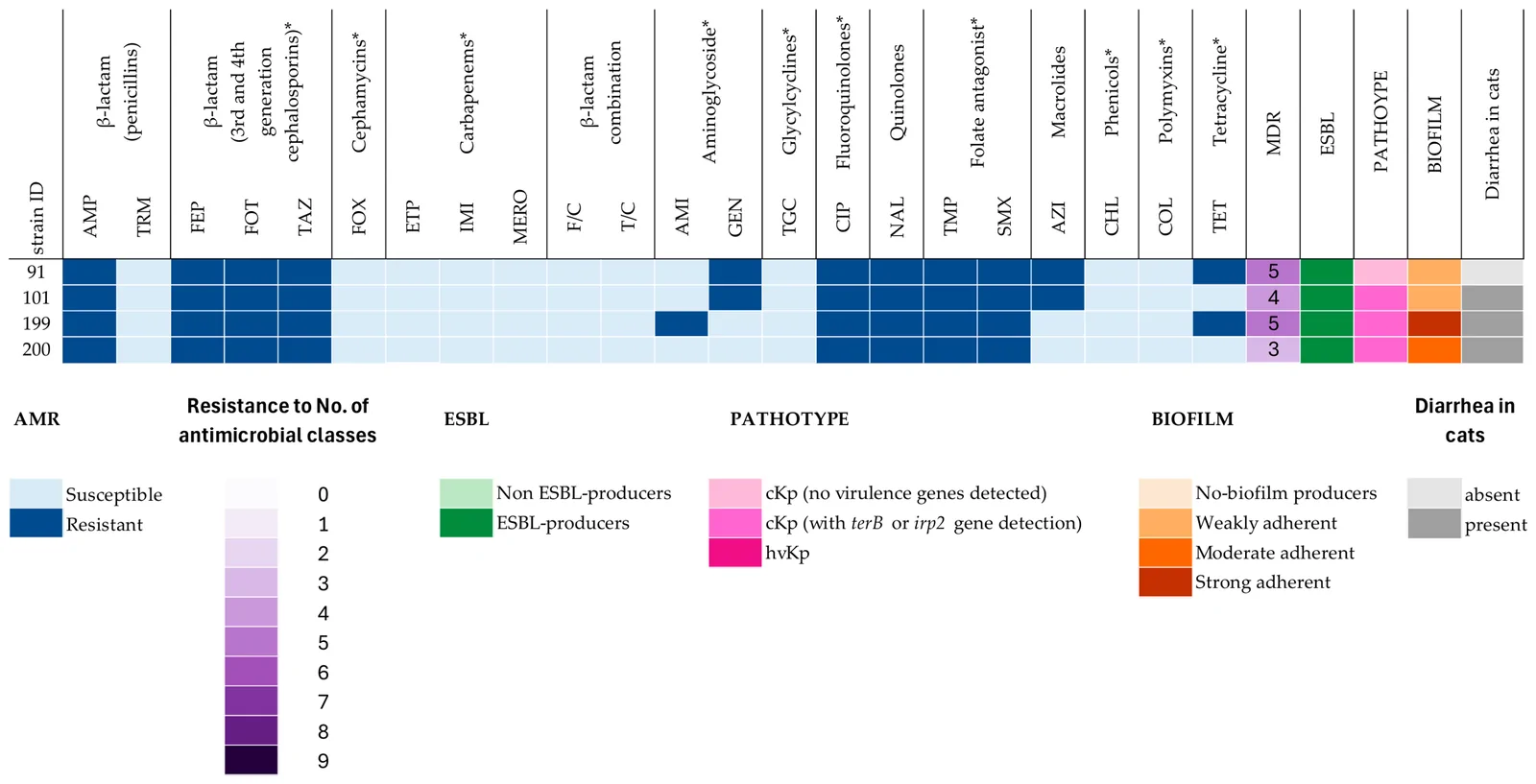
Antimicrobial resistance profile and other characteristics of the four ESBL-producing *K. pneumoniae* complex strains from this study. Individual bacterial isolates are reported in rows. The different antimicrobial agents categorized by class are reported in columns. MDR, ESBL production, pathotype, biofilm formation capacity and diarrhea status in cats are reported in the rightmost columns. *: antimicrobial categories reported to define MDR for Enterobacteriaceae and exclusion of Ampicillin due to intrinsic resistance in *Klebsiella* spp. [22]. AMI: amikacin; AMP: ampicillin; AZI: azithromycin; CIP: ciprofloxacin; CHL: chloramphenicol; COL: colistin; ETP: ertapenem; F/C: cefotaxime/clavulanic acid; FEP: cefepime; FOT: cefotaxime; FOX: cefoxitin; GEN: gentamicin; IMI: imipenem; MERO: meropenem; NAL: nalidixic acid; SMX: sulfamethoxazole; TAZ: ceftazidime; T/C: ceftazidime/clavulanic acid; TET: tetracycline; TGC: tigecycline; TMP: trimethoprim; TRM: temocillin.

Four cats carried *K. pneumoniae* complex isolates based on the results of the preliminary screening using MacConkey agar supplemented with cefotaxime (1 mg/L). According to subsequent phenotypic and molecular analysis, all four isolates tested positive for ESBL production, whereas AmpC- and carbapenemase production were not detected (Figure 1).

Overall, the four ESBL-producing *K. pneumoniae* complex isolates corresponded to 2% (95% CI: 0.06–3.94%) of the 200 fecal samples.

All four ESBL-producing *K. pneumoniae* complex harbored the *bla*_CTX-M_ gene belonging to group 1. Based on sequencing, these genes were all identified as *bla*_CTX-M-15_. All isolates were carriers of the *bla*_SHV_ and *bla*_TEM_ genes.

Presence of the ESBL-producing *K. pneumoniae* complex according to the presence of diarrhea in cats is reported in Figure 1.

### 2.3. Klebsiella pneumoniae Complex Virulence Factors

All four ESBL-producing isolates showed negative string test results, confirmed by the absence of the *peg-344*, *iucA*, *rmpA* and *rmpA2* virulence genes and negative results for the K1 and K2 serotypes. One and two strains harbored the *terB* and *irp2* virulence genes, respectively (Table 2).

The four isolates were defined as classical *K. pneumoniae* complex pathotype (Figure 1).

### 2.4. Antimicrobial Minimum Inhibitory Concentration (MIC)

Figure 1 and Table 3 show the resistance profiles of ESBL-producing *K. pneumoniae* complex isolates based on MIC results.

Based on the MIC results, all four ESBL-producing *K. pneumoniae* complex were MDR according to antimicrobial categories reported to define MDR for Enterobacteriaceae and excluding Ampicillin due to intrinsic resistance in *Klebsiella* spp. [22]. Resistance to the number of antimicrobial classes ranging from three to five is illustrated in Figure 1.

### 2.5. Biofilm Formation

All four ESBL-producing *K. pneumoniae* complex demonstrated biofilm-forming capability. Two strains exhibited weak, one moderate and one strong adhesion. Biofilm-forming profiles are reported in Figure 1.

## 3. Discussion

Our study focused on the identification of ESBL-producing *K. pneumoniae* complex isolates in fecal samples of cats in order to gain some insights into AMR surveillance in companion animals specifically aimed at these bacteria, which have been recognized as the most challenging MDR pathogens with great public health significance [20]. Indeed, *K. pneumoniae* asymptomatically colonizes the skin, mouth, nasopharyngeal, and GI tracts [23], and the GI carriage of *K. pneumoniae* is a major reservoir for infections.

The low positivity rate (2%) of cats harboring ESBL-producing *K. pneumoniae* complex isolates in fecal samples is consistent with previous results, which have showed that ESBL-producing *K. pneumoniae* has been reported in cats and dogs at lower frequencies than ESBL-producing *E. coli* [24]. The few reports that have investigated the prevalence and characterization of ESBL-producing *K. pneumoniae* in fecal samples of pets confirm the 2% positivity observed in our study. More precisely, a study in Guadeloupe (French West Indies) showed 1.7% positivity in cats [24], and another study showed 4.2% positivity in dogs from Portugal [20]. Results from this study are also consistent with the majority of studies focusing on ESBL characterization of clinical *K. pneumoniae* isolates in pets and showing 5.7% positivity (six isolates) in 105 total bacteria previously collected in urine samples of cats and dogs in France [14] and 2.3% positivity in urine samples from dogs in Italy, corresponding to 5.7% positivity when considering the three positive isolates out of the 53 total collected bacteria [13]. Comparison of different studies needs a careful interpretation of results due to geographical differences and different populations studied. In this study, the use of selective enrichment followed by selective culture media may have influenced the detection rate of the ESBL-producing *K. pneumoniae* complex isolates, and differences in isolation protocols should also be taken into account when comparing prevalence estimates across studies. Moreover, considering the collection of a single fecal sample from cats and the possibility that *K. pneumoniae* might transiently colonize pets and lead to intermittent shedding [21], our study may have underestimated the positivity of ESBL-producing *K. pneumoniae* complex isolates.

The presence of *bla*_CTX-M-15_ was expected as this gene has become prevalent in *K. pneumoniae* isolates from humans and companion animals worldwide, including European countries [25]. The presence of *bla*_TEM_ and *bla*_SHV_ genes have been frequently reported in ESBL-producing *K. pneumoniae* [26].

All strains demonstrated multiple drug resistance [22], confirming that *K. pneumoniae* isolates obtained from companion animals often exhibited resistance to different antimicrobials. Besides resistance to β-lactams, including the intrinsic resistance to ampicillin due to the presence of chromosome-borne *bla*_SHV_ genes [26], the resistance of all isolates to fluoroquinolones, quinolones and folate antagonists aligns with previous studies that demonstrated co-existence of various antimicrobial resistances with *bla* genes [18]. In our study, the detection of resistance to tetracycline in two strains is in line with previous investigations showing significant proportions of *K. pneumoniae* isolates from pets demonstrating tetracycline resistance [18]. The low resistance rates of amikacin have already been reported previously [18]. The absence of resistance to carbapenems by MIC analysis aligns with the negative results in the phenotypic characterization to detect carbapenemase-producing bacteria. Overall, the presence of all isolates being MDR aligns with previous reports raising concerns regarding restriction of treatment options and complicating the control of AMR [18]. Moreover, resistance to antimicrobials may present considerable health risk to both humans and animals [27].

The identification of all four strains belonging to classical *K. pneumoniae* and expected as hvKp has been reported in septicemia and systemic illness in pets [28], which were not observed in the cats of this study. The negative string test and the absence of the genetic markers proving a reliable means of distinguishing hvKp from cKp, such as aerobactin siderophore (*iucA*), a putative metabolite transporter (*peg-344*), regulator mucoid phenotypes A and A2 (*rmpA*, *rmpA2*) and the capsular serotypes K1 and K2, confirmed the absence of hvKp isolates from cats included in this study [9,10,29]. On the other hand, the presence of virulence genes such as *terB* and *irp2* in one and two isolates, respectively, was not surprising as these genes have not been defined as accurate markers to identify hvKp isolates [29]. These virulence genes have been reported previously in studies, showing that *terB* contributes to *K. pneumoniae* pathogenicity. More precisely, the *terB* gene has been identified predominantly in digestive and respiratory *K. pneumoniae* strains from ill pets and is among the hypervirulence genes widely detected in respiratory *K. pneumoniae* from humans and companion animals [18]. Moreover, the *irp2* gene has been associated with iron metabolism and facilitates the synthesis of enterobactin, mediating the iron acquisition in Enterobacterales. In ill companion animals, the presence of *irp2* gene has been linked to the high pathogenicity of some *K. pneumoniae* isolates [30]. However, the association of hvKp to severe disease outcomes is derived from human studies. On the other hand, few studies in pets have been performed to identify direct association between virulence genes generally associated with hvKp and increased pathogenicity [28]. The understanding if hypervirulent markers confer the same clinical impact in cats, as observed in humans, needs further investigation. Despite being classified as cKP, further investigations on pathogenicity of the strains identified in this study are also needed to define the potential presence of pathobionts in cats. Pathobionts have been defined as a symbiont that may cause disease in hosts with weakened immune defenses or altered microbiota, and it has been reported that the presence of specific virulence factors in some clones, such as the *irp2* gene found in two isolates of this study, may trigger the GI translocation of the strain to the bloodstream resulting in severe infection [31,32]. In humans, a large proportion of cKp resides as pathobionts inside the body, and approximately half of intensive care unit infections have been attributed to patients’ own *K. pneumoniae* within the GI microbiota [21,33].

The results on the biofilm-forming capacity in ESBL-producing *K. pneumoniae* complex isolates from fecal samples of cats from this study are of concern. Features of virulence and antimicrobial resistance of the isolates further explain the finding of the isolates’ biofilm-forming capacity. Biofilm formation plays a pivotal role in bacterial pathogenicity as biofilms offer increased environmental resilience and serve as key virulence mechanisms. Moreover, biofilm enhances the bacterium’s resistance to antimicrobial agents and external stressors, acting as reservoirs for genetic exchange; therefore, it may promote the spread of antimicrobial resistance [34]. Results from this study further confirm recent evidence indicating that biofilm formation is highly prevalent in ESBL-producing *K. pneumoniae* strains [8]. Considering that biofilms can protect bacteria from both antimicrobial agents and immune defenses [34], results from this study further suggest that therapeutic guidelines for ESBL-producing *K. pneumoniae* infections should not rely solely on antibiotic susceptibility test results but should also incorporate the aspect of biofilm production when optimizing treatment strategies and clinical outcomes [8].

The limited number of ESBL-producing isolates precluded statistical analyses. Consequently, independent risk factors could not be reliably assessed. In this respect, the higher positivity of ESBL-producing *K. pneumoniae* complex strains in cats with diarrhea needs further investigation as *K. pneumoniae* infection not depending on ESBL production has been associated with diarrhea [12,35]. Moreover, the clinical significance of ESBL-producing *K. pneumoniae* complex strains in the three cats with diarrhea needs to be interpreted cautiously because this study did not investigate the causative agent of diarrhea, except ruling out parasitic infections or feline panleukopenia virus etiology (Lauzi S., personal communication). Interestingly, the virulence gene profiles of the three ESBL-producing *K. pneumoniae* complex strains linked to the diarrhea in cats showed positivity for *terB* or *irp2*. However, the understanding of the potential pathogenicity of these strains requires further investigations. On the other hand, the presence of one ESBL-producing *K. pneumoniae* complex strains with negative results for the investigated virulence genes in a fecal sample from a cat with traumatic disease in the absence of diarrhea suggests the colonization of ESBL-producing *K. pneumoniae* complex strains as well as that asymptomatic carriage rather than infection may be present in cats, as reported in some colonization studies of healthy pets [20,21,28]. Cats may contribute to the antimicrobial resistance dissemination and potential zoonotic transmission of AMR isolates.

The presence of ESBL-producing *K. pneumoniae* complex strains observed only in cats treated with antibiotics and hospitalized is not surprising and is consistent with previous studies, indicating that these risk factors are associated with an increase in ESBL-producing *K. pneumoniae* infection [28]. Early documentation of nosocomial *Klebsiella* outbreaks in small animal intensive care units has been reported starting from 1978 [36]. Transmission of multidrug-resistant bacteria is one of the most common causes of nosocomial infections in pets [25,28,36].

Currently, in most countries, AMR surveillance programs primarily focus on humans or livestock. Despite their proximity to humans, pets are rarely included in these surveillance programs. Inclusion of pets would be important, given that the potential transmission of ESBL/AmpC-producing Enterobacterales from companion animals to humans has been reported [11]. Moreover, considering the frequent identification of *bla*_CTX-M-15_ carrying *K. pneumoniae* in human clinical isolates [37], which were also found in samples from cats from this study, the need to integrate companion animal data into One Health AMR monitoring frameworks is further highlighted [28]. AMR monitoring and current infection control strategies have primarily focused on clonal transmission of multidrug-resistant bacteria. However, it is well known that critical AMR genes such as extended spectrum beta-lactamases (*bla*_CTX-M_) in Gram-negative bacteria are frequently carried by plasmids, which can disseminate horizontally across taxonomic boundaries. Although horizontal transfer of plasmids continues to threaten the effectiveness of last-resort antibiotics [38], it is still unclear to which extent plasmid transmission contributes to the overall burden of AMR. Up to now, AMR plasmid transmission routes have neither been understood nor analyzed in the hospital settings. Therefore, further studies should focus on identifying AMR plasmid transmission pathways via the genetic characterization of plasmids of the isolates as previously suggested [38].

Moreover, the results of this study highlight the need for veterinary clinics and hospitals to implement and strengthen infection prevention and control (IPC) measures analogous to those in human healthcare to halt the transmission of ESBL-producing *K. pneumoniae* complex strains and AMR bacteria overall. Prevention of nosocomial outbreaks of MDR pathogens should rely on IPC measures, including appropriate personnel protective equipment (PPE), routine screening of high-risk patients, barrier nursing, and evidence-based disinfections protocols [28].

This study had some limitations. The isolation strategy was intentionally based on selective antibiotic-containing media, resulting in the recovery of only ESBL-producing isolates. Consequently, the study was specifically designed to characterize resistant isolates rather than the overall *K. pneumoniae* population. Therefore, comparisons between ESBL-producing and susceptible isolates with respect to virulence characteristics and biofilm-forming capacity, as well as the evaluation of antimicrobial resistance mechanisms beyond ESBL production, were not performed. This study focused specifically on ESBL-producing *K. pneumoniae* and not on other ESBL-producing Enterobacterales (e.g., *Escherichia coli*) and did not allow for a comprehensive investigation of AMR in fecal samples as well as possible co-carriage of different ESBL-producing bacteria in the same sample. In our study, *K. pneumoniae* complex strains were identified by MALDI-TOF MS, which is not a reliable diagnostic method to discriminate *K. pneumoniae* complex members [39]. Therefore, further identification methods based on whole-genome sequencing (WGS) or sequencing of specific genetic markers are needed to distinguish *K. pneumoniae sensu stricto* from *K. variicola*, *K. quasipneumoniae*. Moreover, novel phylogroups within the complex are needed to understand the true clinical significance of each species and their potential epidemiological specificities. The small number of ESBL-producing *K. pneumoniae* complex isolates and the sampling strategy did not allow us to infer results in the overall population of cats. Furthermore, the absence of a complete clinical and diagnostic procedure did not allow us to understand if the *K. pneumoniae* complex was the cause of the observed clinical status and if other pathogens were involved. This study did not focus on longitudinal sampling of cats to define if the shedding pattern of the ESBL-producing *K. pneumoniae* complex was transient rather than permanent. Furthermore, we did not perform whole-genome sequencing to further characterize the isolates, which would provide higher-resolution data regarding the relatedness of strains, the dissemination of antimicrobial resistance genes, potential transmission pathways, and sequence types typically associated with human clinical strains in order to assess zoonotic potential. Moreover, characterization of susceptible isolates, together with the investigation of resistance mechanisms beyond ESBL production, may provide a more comprehensive understanding of the epidemiology and pathogenic potential of *K. pneumoniae* within the One Health framework.

## 4. Materials and Methods

### 4.1. Animal Selection and Sample Collection

Fecal samples were collected from cats that were admitted between 2020 and 2026 to the Veterinary Teaching Hospital (VTH) of Lodi. Samples were submitted to the VTH of Lodi for diagnostic purposes or as part of health checks following informed consent of the owners. According to the guidelines of the Institutional Animal Care and Use Committee of the University of Milan, the use of residual samples for further analysis was allowed because samples were collected for diagnostic purposes or as part of health checks and with informed consent of the owners (decision No. 2/2016 of the Ethical Committee of the University of Milan).

Data recorded for each animal included sex, age, season of sampling, ownership category, clinical status, hospitalization history and exposure to antimicrobial treatment within the previous three months.

Cats were assigned to one of two age groups (<1 year or ≥1 year) according to previously established criteria. Clinical status was assessed based on clinical examination findings and available laboratory results. Animals presenting signs commonly associated with *K. pneumoniae* infection such as enteric, respiratory, mammary, reproductive and urinary disorders or presence of miscellaneous clinical pictures (including abscesses, otitis, hepatitis, conjunctivitis, pyodermitis, sepsis and encephalitis) [35] were classified as unhealthy, whereas cats without clinical abnormalities or with clinical manifestations not associated with *Klebsiella*-induced infection (e.g., traumatic, neoplastic or other disorders) were classified as healthy/other conditions. Data on the presence of diarrhea was also reported.

Following collection, fecal samples were refrigerated at 4 °C for no longer than four days or frozen at −20 °C for up to one month before processing.

### 4.2. Isolation and Identification of ESBL-Producing K. pneumoniae Complex

This study specifically focused on ESBL-producing *K. pneumoniae* in order to fill in some gaps in the knowledge of its distribution in cats’ fecal samples. Therefore, other ESBL-producing Enterobacterales (e.g., *Escherichia coli*) that were recovered during the screening process were not included in the study and are part of additional investigations. For bacterial isolation, homogenization of one gram of each fecal sample in 9 mL of buffered peptone water (BPW) was carried out. Incubation was performed at 37 °C overnight. A loopful of each enrichment culture was plated on MacConkey agar supplemented with cefotaxime (1 mg/L). Incubation was carried out in aerobiois at 37 °C for 24 h [4].

From each plate, colonies displaying morphology compatible with *Klebsiella* spp. (mucoid and pink lactose-fermenting colonies) were selected for identification. For the identification of bacterial species, the matrix-assisted laser desorption/ionization time-of-flight mass spectrometry (MALDI-TOF MS; MBT Microflex LT/SH, Bruker Daltonik GmbH, Bremen, Germany) was used according to the direct transfer method [40]. Isolates identified by MALDI-TOF MS as *K. pneumoniae* were defined as belonging to the *K. pneumoniae* complex, given the limited discriminatory power of this method among closely related species [39].

All *K. pneumoniae* complex isolates were stored for further bacteriology and molecular analysis. Storage of strains was performed in brain–heart infusion (BHI) broth supplemented with 15% glycerol at −80 °C.

### 4.3. ESBL-/AmpC-/CP-Producing K. pneumoniae Complex Phenotypic Characterization

Phenotypic characterization of *K. pneumoniae* complex isolates was performed according to the recommendations of the European Committee on Antimicrobial Susceptibility Testing (EUCAST) [41], with minor modifications reported by the laboratory protocols defined by the European Union Reference Laboratory for Antimicrobial Resistance [42]. In this respect, to maintain the selective pressure, isolates were thawed, subcultured on MacConkey agar supplemented with cefotaxime (1 mg/L), and incubated overnight at 37 °C. A turbidity equivalent to 0.5 McFarland was obtained, and bacterial suspensions were inoculated onto Mueller–Hinton agar (MH) plates.

The combination disk test (CDT) was used to assess extended-spectrum β-lactamase (ESBL) production according to manufacturer’s instructions (MAST Group Ltd., Bootle, UK). Cefotaxime and ceftazidime disks, alone and in combination with clavulanic acid, were used for ESBL production confirmation.

The AmpC Detection Set D69C and the Carbaplus D73C kit (MAST Group Ltd., Bootle, UK) were used to define AmpC β-lactamases and carbapenemases production, respectively. The Carbaplus assay was used to differentiate *Klebsiella pneumoniae* carbapenemase (KPC), metallo-β-lactamase (MBL), and OXA-48-like carbapenemase producers. Incubation of all plates was performed at 37 °C for 18–24 h according to the manufacturers’ instructions and established protocols. After incubation, inhibition zone diameters were measured and interpreted following EUCAST criteria [41] or the manufacturer’s recommendations, where applicable.

### 4.4. Genetic Characterization of ESBL-Producing K. pneumoniae Complex

Isolates showing a phenotypic profile consistent with ESBL production were subjected to molecular characterization. Genomic DNA was extracted by the boiling method (98 °C for 10 min). The presence of the main ESBL-associated genes was investigated by a polymerase chain reaction (PCR) using a previously described multiplex PCR protocol targeting the *bla*_CTX-M_, *bla*_TEM_ and *bla*_SHV_ genes, with minor modifications [43].

Samples positive for *bla*_CTX-M_ were further analyzed to determine the corresponding *bla*_CTX-M_ group, following a previously published PCR protocol amplifying the *bla*_CTX-M-1_ group [44].

Table 4 shows the sequences of primers used in this study.

In each PCR run, positive control strains and a negative control consisting of DNase-free water were included. Amplicons were separated by electrophoresis on 1.5% agarose gels stained with ethidium bromide and subsequently visualized under UV light.

In the presence of amplicons of the *bla*_CTX-M-1_ group of the expected size, purification of amplicons was performed, and Sanger sequencing was carried out by a commercial sequencing facility (Microsynth Seqlab, Göttingen, Germany) using the same forward and reverse primers used in the DNA amplification. BioEdit software version 7.0 was used to assemble the sequence data. The sequences were then compared with those available in GenBank to identify the *bla*_CTX-M_ type within the *bla*_CTX-M-1_ group.

### 4.5. Phenotypic Detection of Classical and Hypervirulent Types of K. pneumoniae Complex

The string test was used to define the hypervirulent phenotype of *K. pneumoniae* complex isolates (hvKp). The phenotypic presence of hvKp was confirmed by the positive string test result, contributing to the formation of a viscous string measuring ≥5 mm when colonies were stretched with a bacteriological loop following incubation on blood agar plates at 37 °C for 24 h. The classical pathotype of the *K. pneumoniae* complex (cKp) was phenotypically defined by negative results in the string test [29].

### 4.6. Detection of Virulence Genes

Regardless of the phenotypic assessment, molecular identification of cKp and hvKpwas conducted using previously published PCR protocols targeting *peg-344,* aerobactin siderophore biosynthesis *(iucA*), tellurite resistance (*terB*), iron uptake (*irp2*), and regulator mucoid phenotypes A and A2 (*rmpA* and *rmpA2*) virulence genes, with minor modifications [29]. Moreover, specific genes for capsular serotypes K1 and K2 were amplified by PCR, following previously published protocols [45]. The list of primer sequences and PCR product sizes is shown in Table 4.

To ensure accuracy, positive controls and blank controls (DNAse-free water) were included in each PCR run. Amplification products were visualized by gel electrophoresis on 1.5% agarose gels stained with ethidium bromide. Strains were identified as hvKp in the presence of either *peg-344* or *iucA* genes, for which a sensitivity of 0.94 and specificity of 1.0 has been reported [29].

### 4.7. Minimum Inhibitory Concentration (MIC)

In accordance with the guidelines established by the EUCAST, as described in Decision 2020/1729 EU, the broth microdilution method using two commercial plates (Sensititre™ EUVSEC 2 and Sensititre™ EUVSEC 3, Thermo Scientific, Segrate, Milan, Italy) was performed to define the minimum inhibitory concentrations (MICs) of antibiotics for ESBL-producing *K. pneumoniae* complex isolates [3].

Previous to testing, isolates were thawed and subcultured on MacConkey agar supplemented with cefotaxime (1 mg/L) and incubated overnight at 37 °C as previously reported [42]. A 0.5 McFarland concentration was obtained, and bacterial suspensions (ten μL) were mixed in Mueller–Hinton broth (MHB). Fifty μL of MHB containing bacteria were inoculated in the plates. Plates were incubated at 37 °C for 18 h. The MIC values of each antibiotic were defined following reading of results using the Thermo Scientific Sensititre™ Manual Viewbox. To interpret the MIC values, EUCAST human clinical breakpoints were used [46]. EUCAST ECOFFs [47] or CLSI M100 [48] were used in the absence of available EUCAST human clinical breakpoints.

Based on the MIC results, isolates showing resistance to at least one agent in three or more antimicrobial categories were classified as multidrug-resistant (MDR) according to the antimicrobial categories reported previously to define MDR for Enterobacteriaceae and excluding Ampicillin due to intrinsic resistance in *Klebsiella* spp. [22].

### 4.8. Biofilm Formation

To assess biofilm formation, a microtiter plate (MtP) assay was used, as previously described [49]. Briefly, *K. pneumoniae* complex isolates were grown in BHI broth for 24 h at 37 °C and subsequently diluted at a ratio of 1:100 in Tryptone Soy Broth (TSB) supplemented with 1% glucose. Bacterial suspension was inoculated in 96-well plates. Plates were incubated for 24 h at 37 °C. Following incubation, planktonic bacteria were removed by gentle washing of the wells, which was performed three times. Biofilm was fixed with 99% methanol and stained with Crystal Violet solution. In order to remove the excess stain from the wells, a washing step was performed, and the biofilm-bound crystal violet was then solubilized using 70% ethanol. A negative control was used, represented by TSB + 1% glucose only. Analysis of each isolate was done in triplicate on the same plate, and three independent plates were used. The optical density (OD) of each well was measured by reading at 570 nm using an Epoch ELISA reader (BioTek Instrumentals, Winooski, VT, USA). The average OD of the negative control was calculated, and three standard deviations (SDs) of the negative control were added to the average OD value of the negative control to determine the optical density cut-off (ODc). Biofilm formation of the isolates was categorized based on the OD of the sample, as previously reported. Samples were identified as not, weakly, moderately, and strongly adherent [49].

### 4.9. Sample Size and Data Analysis

WinEpi (http://www.winepi.net/uk/index.htm, accessed on 1 October 2025) was used for sample size calculation to detect the presence of ESBL-producing *K. pneumoniae* complex strains in cats, considering a population size of 500 cats admitted to the VTH per year for routine diagnostic analysis on fecal samples, a minimum prevalence of 1.5%, and 95% confidence intervals. A total sample size of 200 animals was calculated.

The 95% confidence limits were calculated for ESBL-producing *K. pneumoniae* complex-positive animals.

## 5. Conclusions

In conclusion, this investigation provides insights into the occurrence and characteristics of ESBL-producing *K. pneumoniae* complex isolates obtained from the fecal samples of cats in Italy. The presence of ESBL genes and biofilm-forming capacity along with the detection of some isolates possessing virulence factors suggests that cats may act as potential reservoirs of antimicrobial-resistant *K. pneumoniae* complex strains that could spread to other animals and potentially humans and the environment. This warrants further investigations under the One Health concept and highlights the need for regular surveillance in veterinary clinics, enforcing stewardship and fostering cross-sectional collaboration at both local and global levels to detect, prevent, and control the dissemination of the ESBL-producing *K. pneumoniae* complex at the animal–human–environmental interface.

## Figures and Tables

**Table 1:** Characteristics of cats analyzed in this study, and presence of ESBL-producing *K. pneumoniae* complex.

Population Characteristics		No. (%)	ESBL-Producing *K. pneumoniae* Complex No. (%)
Sex	Male	107 (53.5)	2 (1.9)
	Female	93 (46.5)	2 (2.2)
Age ^1^	<1 year	66 (33)	0
	≥1 year	128 (64)	4 (3.1)
Type of ownership	Privately owned	62 (31)	0
	Stray/shelter	138 (69)	4 (2.9)
Clinical status	Healthy/other conditions	134 (67)	1 * (0.8)
	Unhealthy	66 (33)	3 (4.6)
Diarrhea presence	Yes	31 (15.5)	3 (9.7)
	No	169 (84.5)	1 * (0.6)
Gastrointestinal symptoms	Yes	40 (20)	3 (7.5)
	No	160 (80)	1 * (0.6)
Hospitalization	Yes	68 (34)	4 (5.9)
	No	132 (66)	0
Antibiotic therapy ^2^	Yes	59 (29.5)	4 (6.8)
	No	103 (51.5)	0

^1^ Age was unknown for six cats. ^2^ Antibiotic therapy was unknown for 38 cats. *: cat n. 91 showing traumatic disease in absence of diarrhea.

**Table 2:** String test and virulence genes results in ESBL-producing *K. pneumoniae* complex isolates detected in this study.

Strain ID	String Test	*peg-344*	*iucA*	*rmpA*	*rmpA2*	*terB*	*irp2*	K1 (*MagA*)	K2 (*wzy*)
91	-	-	-	-	-	-	-	-	-
101	-	-	-	-	-	+	-	-	-
199	-	-	-	-	-	-	+	-	-
200	-	-	-	-	-	-	+	-	-
Total		-	-	-	-	1	2	-	-

**Table 3:** Number of *K. pneumoniae* complex isolates resistant to antimicrobial agents based on MIC clinical breakpoint (µg/mL) reported by EUCAST.

Antimicrobial Class	Antimicrobial Agent	Breakpoint	Total No. Resistant/Total Analyzed (%)
B-lactam (penicillins)	AMP	8	4/4 (100%)
	TRM	16	0
B-lactam (3rd and 4th generation cephalosporins)	FEP	4	4/4 (100%)
	FOT	2	4/4 (100%)
	TAZ	4	4/4 (100%)
Cephamycins	FOX	8	0
Carbapenems	ETP	0.5	0
	IMI	4	0
	MERO	8	0
B-lactam combination	F/C	0.25 ^1^	0
	T/C	0.5 ^1^	0
Aminoglycoside	AMI	8	1/4 (25%)
	GEN	2	2/4 (50%)
Glycylcyclines	TGC	2 ^1^	0
Fluoroquinolones	CIP	0.5	4/4 (100%)
Quinolones	NAL	≥32 ^2^	4/4 (100%)
Folate antagonist	TMP	2	4/4 (100%)
	SMX	0.5	4/4 (100%)
Macrolides	AZI	16	2/4 (50%)
Phenicols	CHL	16	0
Polymyxins	COL	2	0
Tetracycline	TET	≥16 ^2^	2/4 (50%)

^1^ EUCAST MIC ECOFF (μg/mL) was used. ^2^ Human CLSI MIC clinical breakpoint (µg/mL) was used. AMI: amikacin; AMP: ampicillin; AZI: azithromycin; CHL: chloramphenicol; CIP: ciprofloxacin; COL: colistin; ETP: ertapenem; F/C: cefotaxime/clavulanic acid; FEP: cefepime; FOT: cefotaxime; FOX: cefoxitin; GEN: gentamicin; IMI: imipenem; MERO: meropenem; NAL: nalidixic acid; SMX: sulfamethoxa-zole; TAZ: ceftazidime; T/C: ceftazidime/clavulanic acid; TET: tetracycline; TGC: tigecycline; TMP: trimethoprim; TRM: temocillin.

**Table 4:** Primers used in this study.

Target	Primer	Sequence 5’-3’	Length (bp)	Reference
CTX-M	*bla*_CTX-M_ F	ATGTGCAGYACCAGTAARGTKATGGC	593	[43]
	*bla*_CTX-M_ R	TGGGTRAARTARGTSACCAGAAYCAGCGG		
CTX-M-1 group	*bla*_CTX-M-1_ F	GGTTAAAAAATCACTGCGYC	865	[44]
	*bla*_CTX-M-1_ R	TYGGTGACGATTTTAGCCGC		
TEM	*bla*_TEM_ F	TCGCCGCATACACTATTCTCAGAATGA	445	[43]
	*bla*_TEM_ R	ACGCTCACCGGCTCCAGATTTAT		
SHV	*bla*_SHV_ F	ATGCGTTATATTCGCCTGTG	747	[43]
	*bla*_SHV_ R	TGCTTTGTTATTCGGGCCAA		
*peg-344*	*peg-344* F	AAAGGACAGAAAGCCAGTG	411	[29]
	*peg-344* R	CAATGACGAGGGGGATAATC		
*iucA*	*iucA* F	AATCAATGGCTATTCCCGCTG	239	[29]
	*iucA* R	CGCTTCACTTCTTTCACTGACAGG		
*terB*	*terB* F	TATCGCTGTTGCCAGTGAC	288	[29]
	*terB* R	CGGACAGCACTCTTCTCATC		
*irp2*	*irp2* F	GCTACAATGGGACAGCAACGAC	230	[29]
	*irp2* R	GCAGAGCGATACGGAAAATGC		
*rmpA*	*rmpA* F	GAGTAGTTAATAAATCAATAGCAAT	332	[29]
	*rmpA* R	CAGTAGGCATTGCAGCA		
*rmpA2*	*rmpA2* F	GTGCAATAAGGATGTTACATTA	430	[29]
	*rmpA2* R	GGATGCCCTCCTCCTG		
K1 (*MagA*)	*MagA* F	GGTGCTCTTTACATCATTGC	1283	[45]
	*MagA* R	GCAATGGCCATTTGCGTTAG		
K2 (*wzy*)	*wzy* F	GACCCGATATTCATACTTGACAGAG	641	[45]
	*wzy R*	CCTGAAGTAAAATCGTAAATAGATGGC		

## Data Availability

The data that supported the findings of this study are available from the corresponding author upon reasonable request.

## Abbreviations

The following abbreviations are used in this manuscript:

3GC	Third-generation cephalosporin
AMI	Amikacin
AMP	Ampicillin
AMR	Antimicrobial resistance
AZI	Azithromycin
BHI	Brain–heart infusion
BPW	Buffered peptone water
CDT	Combination disk test
CHL	Chloramphenicol
C.I.	Confidence interval
CIP	Ciprofloxacin
cKp	Classical *K. pneumoniae*
CLSI	Clinical and Laboratory Standards Institute
COL	Colistin
CP	Carbapenemase
ECOFF	Epidemiological cut-off value
ESBL	Extended spectrum β-lactamase
ESKAPE	*Enterococcus faecium*, *Staphylococcus aureus*, *K. pneumoniae*, *Acinetobacter baumannii*, *Pseudomonas aeruginosa*, and *Enterobacter* species
ETP	Ertapenem
EUCAST	European Committee on Antimicrobial Susceptibility Testing
F/C	Cefotaxime/clavulanic acid
FEP	Cefepime
FOT	Cefotaxime
FOX	Cefoxitin
GEN	Gentamicin
GI	Gastrointestinal
hvKp	Hypervirulent *K. pneumoniae*
IMI	Imipenem
IPC	Infection prevention and control
KPC	*Klebsiella pneumoniae*—carbapenemase
MALDI-TOF MS	Matrix-assisted laser desorption/ionization time-of-flight mass spectrometry
MBL	Metallo-β-lactamase
MDR	Multidrug resistant
MERO	Meropenem
MHB	Mueller–Hinton broth
MIC	Minimum inhibitory concentration
MtP	Microtiter plate
NAL	Nalidixic acid
ODc	Optical density cut-off
OXA	Oxacillinase
PCR	Polymerase chain reaction
PPE	Personnel protective equipment
SMX	Sulfamethoxazole
TAZ	Ceftazidime
T/C	Ceftazidime/clavulanic acid
TET	Tetracycline
TGC	Tigecycline
TMP	Trimethoprim
TRM	Temocillin
TSB	Tryptone Soy Broth
VTH	Veterinary Teaching Hospital
WHO	World Health Organization
